# Omics Data Preprocessing for Machine Learning: A Case Study in Childhood Obesity

**DOI:** 10.3390/genes14020248

**Published:** 2023-01-18

**Authors:** Álvaro Torres-Martos, Mireia Bustos-Aibar, Alberto Ramírez-Mena, Sofía Cámara-Sánchez, Augusto Anguita-Ruiz, Rafael Alcalá, Concepción M. Aguilera, Jesús Alcalá-Fdez

**Affiliations:** 1 Department of Biochemistry and Molecular Biology II, University of Granada, 18071 Granada, Spain; 2 "José Mataix Verdú" Institute of Nutrition and Food Technology (INYTA), Center of Biomedical Research, University of Granada, 18100 Granada, Spain; 3 Biosanitary Research Institute of Granada (IBS.GRANADA), 18012 Granada, Spain; 4 Centre for Genomics and Oncological Research (GENYO), 18016 Granada, Spain; 5 Department of Computer Science and Artificial Intelligence, Andalusian Research Institute in Data Science and Computational Intelligence (DaSCI), University of Granada, 18071 Granada, Spain; 6 Barcelona Institute for Global Health (ISGlobal), 08003 Barcelona, Spain; 7 CIBER Physiopathology of Obesity and Nutrition (CIBEROBN), Instituto de Salud Carlos III, 28029 Madrid, Spain

**Keywords:** machine learning, omics, data pre-processing

## Abstract

The use of machine learning techniques for the construction of predictive models of disease outcomes (based on omics and other types of molecular data) has gained enormous relevance in the last few years in the biomedical field. Nonetheless, the virtuosity of omics studies and machine learning tools are subject to the proper application of algorithms as well as the appropriate pre-processing and management of input omics and molecular data. Currently, many of the available approaches that use machine learning on omics data for predictive purposes make mistakes in several of the following key steps: experimental design, feature selection, data pre-processing, and algorithm selection. For this reason, we propose the current work as a guideline on how to confront the main challenges inherent to multi-omics human data. As such, a series of best practices and recommendations are also presented for each of the steps defined. In particular, the main particularities of each omics data layer, the most suitable preprocessing approaches for each source, and a compilation of best practices and tips for the study of disease development prediction using machine learning are described. Using examples of real data, we show how to address the key problems mentioned in multi-omics research (e.g., biological heterogeneity, technical noise, high dimensionality, presence of missing values, and class imbalance). Finally, we define the proposals for model improvement based on the results found, which serve as the bases for future work.

## 1. Introduction

In recent years, the biomedical field has experienced a big data revolution. Since the appearance of the first microarray technologies, the competencies of generating data and extracting useful knowledge have increased exponentially. In fact, now we can perform a whole range of molecular analyses on a genome-wide scale, generally referred to as omics analyses. Omics technological advances have led to breakthroughs in our fundamental understanding of cell biology: from our ability to identify alterations in the DNA sequence via a genome-wide association study (GWAS), to the study of gene expression levels by means of RNAseq experiments, or the possibility of studying environmentally inducible chemical DNA modifications with an epigenome-wide association study (EWAS). Similarly, omics studies are generating very positive insights into improving our knowledge of clinical treatments and managing multi-factorial and complex diseases. One of the most promising clinical applications of omics technologies has been the generation of predictive biomarker panels for personalized estimations of disease risk and the consequent implementation of stratified clinical guidelines. In this regard, omics technologies have taken further advantage of the recent advances in the machine learning (ML) field. ML is a research branch of artificial intelligence that has experienced a notable boost due to its ability to automatically generate predictive and descriptive models from massive amounts of data. Within the context of predictive modeling, increasingly sophisticated ML algorithms have become available; highlighting ensemble modeling or the recent revolution of deep learning [1]. We can highlight some promising examples of biomedical applications of ML models, such as predicting the glycemic response from food intake [2] or the response to treatment in breast cancer patients [3]. In addition, massive omics data generation has been used with ML tools to reconstruct the 3D structure of the genome, model chromatin state, identify genes/regulatory elements, predict the relationship between promoters/enhancers and gene expression, predict transcription factor binding sites, predict protein–protein interactions, stratify metabolic phenotypes, and diagnose/classify different diseases [4]. However, experts in omics data analysis are often unaware of the assumptions behind machine learning models violating some of them. For this, it is relevant to avoid pitfalls in biological studies, such as not differentiating training/test data, not including the confounding variables in the model, and not treating class imbalance. These pitfalls are described in more detail elsewhere [5]. Regardless of the aforementioned potential applications and benefits that the fields of omics and ML yield, the main Herculean task is to translate these promises into tangible predictive models in daily clinical practice [6].

Most of the challenges encountered are related to the implementation of accurate and reliable analytic pipelines, which are aggravated by the shortage of suitably trained professionals to perform such complex data analysis tasks. This is mainly due to the complex nature of omics data, with huge variations across platforms, different needs for pre-processing steps, intense heterogeneity within and between human subjects, and the ubiquitous problem of high-dimensionality and low sample size settings. Predictive modeling is severely affected by high dimensionality due to what is known as the curse of the dimensionality problem. Some advanced pre-processing methods for feature selection, such as ridge regression, lasso, and elastic-net, have been used to improve the performances of ML models. Research shows that these techniques, despite their great potential, are not recommended for use with low data sample sizes because they cause overfitting [7,8]. In this sense, the principal component analysis (PCA) is used in several studies, but its use could involve a loss of interpretability and/or biological meaninglessness. Other techniques, such as filters and wrappers, can be valid solutions as long as they are accompanied by validation to confirm the biological sense. This scenario encourages the creation of new feature selection methods for omics data with very low sample sizes. In this paper, we proposed the realization of feature selection based on human expert knowledge with as much biological sense as possible. Once non-sense features are eliminated, automatic feature selection is perfectly handled by the applied ML method, since all of them are well-known and recognized algorithms, including feature selection as an important part of their learning process [9].

The selection of the most suitable pre-processing pipeline for each omics layer and the choice of the most appropriate ML model are critical steps that must take place considering the particularities of human datasets and depending on the purpose of each predictive modeling tool. This problem increases if we take into account the need to create interpretable models. To address this need, the recent explainable artificial intelligence (XAI) revolution has emerged, which recommends the use of transparent models that are easily understood by human users or the use of post hoc mechanisms that provide comprehensibility to models that are not understandable by scientists, which is especially relevant for medical applications [10]. In the present paper, we reviewed some of the particularities that make predictive modeling with multi-omics data a challenging task and propose adequate solutions that are currently employed in ML-omics research. In order to illustrate the process, we present a case study based on the generation of a predictive ML model following a longitudinal design in children with obesity and metabolic dysfunction. In this population, a series of multi-omics data layers (GWAS and EWAS), as well as biochemical and clinical variables, were available at the pre-pubertal stage. In addition, the metabolic status reached by each child at the pubertal stage was determined by the presence of insulin resistance (IR). The main objective of the contribution was the construction of a robust ML predictive model capable of predicting the IR status of each child by analyzing multi-omics and biochemical pre-pubertal data. In this paper, we describe the main challenges faced by omics ML predictive modeling and propose specific data pre-processing guidelines and different analytical solutions to these challenges. Furthermore, we described the rationale and recommendations that should guide the selection of an ML algorithm and experimental design using a case study of childhood obesity as an example [9].

## 2. Materials and Methods

### 2.1. Description of Case Study Population and Data

**Figure 1:**
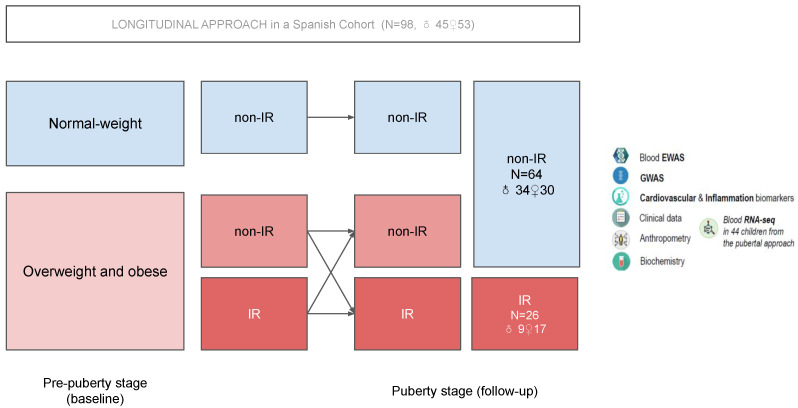
Summary of the PUBMEP project.

The PUBMEP (“PUBberty and Metabolic risk in obese children. Epigenetic alterations and Pathophysiological and diagnostic implications”) project is a longitudinal research study in which children with and without obesity are followed from pre-puberty to puberty evaluating the prevalence of metabolic syndrome and the progression of related cardiometabolic risk factors. In this population, a series of multi-omics analyses were conducted with the aim of discovering new and promising blood molecular biomarkers of IR during the metabolically critical period of puberty (see Figure 1) [11].

IR is one of the metabolic alterations derived from obesity that appears the earliest in patients. If not properly addressed, IR finally results in the development of more severe diseases, such as cardiovascular disease or type II diabetes. For this reason, IR has become a cornerstone in preventing obesity-associated morbimortality. In the PUBMEP study, 90 Spanish children (47 females) were allocated into two experimental groups according to their IR status (IR or non-IR) after the onset of puberty (see Figure 1). The number of children with respective gender distribution in each group can be found in Figure 1. In this population, as mentioned in the introduction, pre-pubertal ($T_{0}$) data (GWAS, EWAS, clinical, anthropometric, and biochemistry) were employed as predictors for the IR status at the pubertal stage ($T_{1}$). For this purpose, several pre-processing steps and ML models were implemented as detailed below. In the current paper, datasets were divided into GWAS, EWAS, and biochemistry (which also incorporated data from anthropometry and clinical history). Children from the PUBMEP project were recruited in three different Spanish cities: Santiago de Compostela, Zaragoza, and Córdoba. As detailed below, the recruitment origin was considered a substantial source of confounding, and was, therefore, taken into account during the analyses. An extensive description of the PUBMEP project can be found elsewhere [11].

### 2.2. Data Pre-Processing Guidelines and Analytical Assessment of ML Predictive Models

#### 2.2.1. GWAS Data

Genomic data were generated by the sequencing of blood samples using a bead chip called Infinium Global Screening-24-v3.0. This technology allowed us to measure 651,563 single nucleotide polymorphisms (SNPs) with a small percentage of missing values. Genotype imputation was performed using the Minimac 4 method from the haplotype reference consortium (HRC) reference (GRCh37/hg19 genomic annotation) panel using the Michigan imputation server [12]. On this server, an automated quality control analysis was performed prior to imputation. The exclusion criteria for this initial quality control were the standard criteria defined by this software: low allelic frequency (less than 0.2); low call rate (less than 0.95); and repeated variants or without information. Once our genomic data were imputed, a second quality control analysis was performed with PLINK 1.9 software [13]. The second quality control exclusion criteria were: low imputation quality ($R^{2}<0.9$); variants that did not meet the Hardy–Weinberg equilibrium ($HWE-P>10^{-6}$); and low minor allele frequency (MAF<0.01) [14].

The underlying bases of the inheritance of diseases were not always the same (e.g., autosomal dominant, recessive, or co-dominant); this has direct effects on the way we represent data and construct predictive ML models. In the case of obesity, as mentioned in the previous section, we are dealing with a complex trait with a strong polygenic and additive nature (the accumulation of many low-risk effects of SNPs is what constitutes a high-risk profile). Considering this, GWAS data were encoded according to the additive model in this paper. For this reason, we propose using a dosage format (raw) to perform the classification task. A dosage format indicates the presence or absence of a risk or reference allele in a SNP encoded with 0, 1, or 2. One advantage of its use is that it allows the use of numerical genetic variables, making it suitable for the algorithm’s learning process [13,15].

Regarding feature selection prior to ML application, we selected a subset of 151 SNPs from the entire array according to previous evidence in the literature. In particular, we collected SNPs highlighted in the meta-analysis, since they are considered to be the studies with the highest degrees of evidence, guaranteeing high statistical power to detect the small effects that each SNP could exert on the phenotype. For this purpose, we performed a literature and database search (GWAS and PGS catalog [accessed on 25 June 2022]; https://www.ebi.ac.uk/gwas/, https://www.pgscatalog.org/, [16,17]) and selected three articles that performed meta-analyses on large populations of European descent [18,19,20].

(1) $$\beta =\frac{M}{M+U},$$

(2) $$M=log_{2}\left(\frac{M}{U}\right),$$

#### 2.2.2. EWAS Data

**Figure 2:**
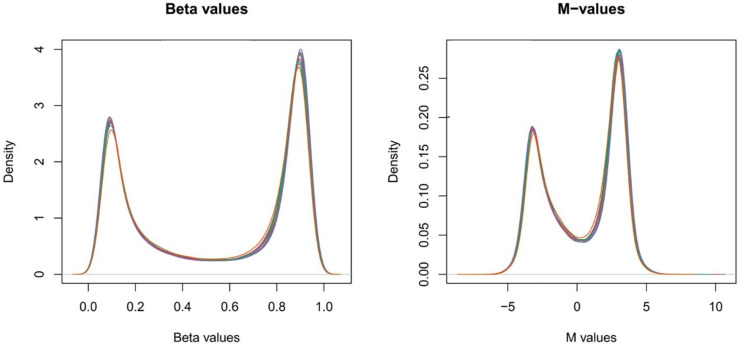
Comparison between β and M values. This image was taken and modified from [23].

EWAS data were generated using the Infinium MethylationEPIC 850K from blood samples. To remove any source of technical variability, low-performing probes were filtered out according to different criteria: probes with a detection *p*-value above 0.01 in more than 10% of the samples, probes with SNPs, cross-reactive probes that aligned to multiple locations, and probes located on the Y chromosome. Regarding normalization, we applied Beta-Mixture Quantile (BMIQ) normalization, which affects only biased type II probes, using the wateRmelon R package. The selection of this normalization method was based on the fact that all samples under study were obtained from the same tissue (blood) [21]. Regardless of the Illumina microarray version employed, for each CpG, there are two measurements: a methylated intensity (denoted by M) and an unmethylated intensity (denoted by U). These intensity values can be used to determine the proportion of methylation at each CpG locus. Methylation levels are commonly reported as either β values or M-values (see Equations (1) and (2); as well as Figure 2). A detailed comparison of M-values and β values is available elsewhere [22]. Lastly, we obtained the β and M values of 834,371 CpG sites.

In this case, the feature-selection procedure consisted of the application of an agnostic selection, a type of feature selection in which differentially methylated CpG sites associated with IR were extracted genome-wide (hypothesis-free). This procedure was conducted in an independent population study that had the same origins as our study population, with some samples overlapping. The study population, which facilitated the agnostic selection, was part of a study of 139 children (76 girls), including longitudinal and cross-sectional approaches, and followed the same experimental design. More details about the selection of these CpG sites can be found in reference [24]. The choice of performing an agnostic selection for the phenotype of interest (IR) instead of relying on the literature findings in GWAS data was motivated by the fact that epigenetics findings are strongly conditioned by the characteristics and environmental exposures of each population. In this regard, having an independent sample with the same characteristics as the current study cohort was a better option than selecting CpG sites according to European population studies (among which, child studies are scarce) [9].

#### 2.2.3. Biochemistry, Anthropometrical, and Clinical Data

The last dataset is referenced as the biochemistry dataset; it involves the combination of data of diverse origins as mentioned previously. This dataset consists of 48 input variables related to the pubertal IR problem. The main problem with these data involved the presence of missing values. The structures of missing data in our cohort were checked (missing completely at random (MCAR), missing at random, missing not at random, and structurally missing). Then, 14 biochemical variables with more than 5 missing values were discarded in order to avoid introducing excessive noise into the data via imputation. We revised several imputation methods, such as mean/median imputation, knn imputation, bagged trees, Multiple imputations by chained equations (MICE) [25], and missForest [26]. We chose the missForest method for several reasons: it is a non-parametric method that can impute continuous and categorical features, does not require tuning parameters because of its robust performance, and does not require assumptions about the distribution of the features. This method was used in the final 34 features via the missForest R package [26].

### 2.3. Basis and Recommendations That Must Guide the Selection of a ML Algorithm and the Experimental Design

#### 2.3.1. Experimental Design

After completing individual pre-processing procedures, three different datasets (GWAS, EWAS, and biochemistry) were obtained. Each dataset had 1 response variable with 2 distinct classes (IR and non-IR) out of 90 children. The input feature numbers (per dataset) were 151, 267, and 34 for the GWAS, EWAS, and biochemistry data, respectively. A summary of the main characteristics of each dataset considered in this study, as well as the number of variables fulfilling quality filters can be seen in Table 1.

Although a promising approach would have involved the simultaneous modeling of several omics layers together with biochemistry data, merging so much information into a single model would also increase the problem of high dimensionality. Moreover, the different nature of each dataset makes it essential to take a first look at the models constructed separately, in order to understand the amount of valuable information available in each source. In this paper, as a preliminary approach, we propose generating independent ML predictive models for each layer of data, leaving multi-omics modeling as a pending task for future work. Our approach allowed us to extract predictive information from the different biological layers and validate the most important variables for the IR problem while avoiding overfitting [9].

One of the most important practices in the ML field is to train the algorithms on a set of individuals differing from the set aimed to evaluate the model performance. If it is not possible to access an independent population, then the training and test sets must be selected iteratively from the same population through a process known as cross-validation (CV). There are several types of CV: leave one out (LOOCV), Montecarlo CV, Bootstrap, k-fold CV, and repeated k-fold CV. Generally, the default k-fold CV is preferred because it presents the average estimations with the least possible errors. Choosing the right validation methodology (according to the characteristics of the data) is the key to preventing erroneous conclusions from the models [27].

Another important factor is that the learning process should be as homogeneous as possible in each iteration. That is, the distribution of the variables and the proportion of classes should be the same in the training and test sets for each iteration of the CV process. In this paper, a stratified 5-fold CV, repeated 5 times, was used to evaluate the model performance, adding up to a total of 25 executions. Research shows that this approach is one of the best CV procedures to reduce the variability of average classification metrics in low sample size designs. Although other CV methodologies such as LOOCV have also been commonly used in the context of low sample sizes, we continue to recommend the use of repeated k-fold cross-validation for similar studies where the sample size is low, as this methodology has the lowest estimation error, offers a good bias-variance ratio, and is a computationally affordable procedure [27,28].

As can be seen in Figure 1, the datasets from the case study present a severe class imbalance that could lead to overfitting in terms of the majority and minority classes. With this in mind, oversampling and undersampling techniques were tested on the training sets to “balance” the learning procedure while keeping the original samples and distributions for the test sets. The resampling method employed was the default method from the R package ’themis’ [29]. To confirm that learning occurs equally in both classes, it is necessary to evaluate the performances of the models by looking at different classification metrics [30].

#### 2.3.2. Selection of ML Algorithms and Classification Metrics

Another point of debate when constructing a predictive model is the choice of the ML algorithm and the metrics to be used, which will be strongly conditioned by the objective to be pursued. For example, looking for a model with high predictive ability, neural networks, support vector machines, random forests, or boosted trees might be valuable options. However, if a model is to be used in clinical practice, clinicians must understand how the algorithm makes decisions due to the ethical issues underlying decision-making that may have an impact on the patients’ lives. In such cases, we may opt for more interpretable models, such as decision trees or other rule-based methods, avoiding the so-called black box models, whose predictions lack understandable explanations of their underlying internal mechanisms [31]. The need to find models that provide both good predictive performances and explainability has recently increased the popularity of XAI, leading to the use of comprehensive models or the development of techniques that provide explainability to such models [10]. In this regard, techniques (e.g., the SHapley Additive Explanations (SHAP) feature attribution framework) were developed to provide explainability to models whose internal behaviors are not directly understandable due to their complexities [32,33].

In this study, we chose the well-known OneR [34], CART [35], and XGBoost [36] algorithms for the purpose of showing the behaviors of various ML methods that provide transparent classifiers over the different omics datasets, because it is essential for experts to understand how the models make their predictions. OneR and CART generate transparent classifiers based on a single-rule system and a simple decision tree, respectively, so the experts can understand them directly due to their nature. On the other hand, XGBoost generates an accurate ensemble-type classifier using gradient boosting. This classifier has become very popular because of its ability to achieve good performance results on structured data. However, this algorithm provides a black-box classifier based on a tree ensemble, involving the need to use SHAP explanations to understand the generated model.

There are several metrics of interest when evaluating the performance of a model. Metrics that consider classes separately, such as sensitivity or specificity, provide valuable information about that class but should be complemented with other measures to obtain a complete picture of the model’s behavior. Some performance indicators combine several metrics in an effort to achieve a more comprehensive approach, such as G-mean—the geometric mean of sensitivity and specificity—which provides an assessment of how well-balanced the sensitivity and specificity values are. Balancing the accuracy between the majority and minority classes is the key to avoiding underfitting the minority class while overfitting the majority class. Of course, the metrics used to evaluate the model are strongly conditioned by the problem being addressed, e.g., in cases where correctly predicting the positive class is critical, we should focus more on sensitivity than specificity, or make use of metrics that especially penalize failures in the prediction of the positive class, such as G-mean and F1 [30].

All trained models are included in the caret [37] package (classification and regression training) available for R [38] and the parameters were tuned in order to optimize the model’s performance. F1, AUC, G-mean, accuracy, sensitivity, and specificity metrics were considered to evaluate the models. Then, different models were trained for the GWAS, EWAS, and biochemistry datasets using both the original (and imbalanced) datasets and the balanced versions of the original datasets, after being undersampled via the nearmiss method included in the themis package, after optimizing its neighbor’s parameter.

#### 2.3.3. SHAP Explanations

One of the drawbacks to using complex but powerful methods, such as XGBoost, lies in the fact that its behavior is difficult for humans to understand. SHAP helps to understand the mechanisms behind its decisions, using the concept of the Shapely value, which goes back to game theory and can help to understand every single decision made by the model by assigning an attribution to each variable, the sum of which equals the model output for that specific instance. We can think of our model as a game in which every variable plays a role to obtain the model’s output, which can be computed as the sum of the individual attribution (with sign and magnitude) of each variable.

We also use SHAP to compute the overall importance of each variable considering the whole dataset, so that we can obtain a global idea of the role in the insulin resistance mechanism. Equation (3) shows how $I_{j}$, the importance for any predictor j, can be calculated as the mean of the absolute Shapley value of variable j for every sample i, denoted by $|\phi_{j}^{\left(i\right)}|$.

(3) $$I_{j}=\frac{1}{n}\sum_{i=1}^{n}\left|\phi_{j}^{\left(i\right)}\right|$$

## 3. Results

In this study, we illustrated how to face the main challenges related to ML prediction in the multi-omics analysis, using a case study on childhood obesity. Our multi-omics dataset is composed of data from GWAS, EWAS, and biochemistry. From the GWAS, we initially had 651,563 SNPs that were matched to HRC, subjected to the initial quality control, imputed in the Michigan Imputation Server, and subjected to a final quality control analysis. The remaining 5,894,726 SNPs fulfilled the eligibility criteria. Then, a feature selection of SNPs was performed before the ML application in order to select a subset of the total 151 SNPs, of which there was prior knowledge about their association with obesity. Consequently, the EWAS dataset was also subjected to quality control, resulting in the elimination of 56,478 low-performing probes and the remaining 834,371 CpG sites. Next, the feature-selection procedure was applied based on an agnostic selection and we selected a final number of 267 CpG sites.

When we addressed the class imbalance, the quality of all classifiers improved, as shown in Table 2. Furthermore, the classifiers that used the biochemical data layer obtained the best results, followed by those generated with the EWAS and GWAS datasets, respectively. It is worth noting that the XGBoost classifiers achieved the highest values of the G-mean metric, presenting robust performances on all datasets (0.60, 0.62, 0.64).

**Figure 3:**
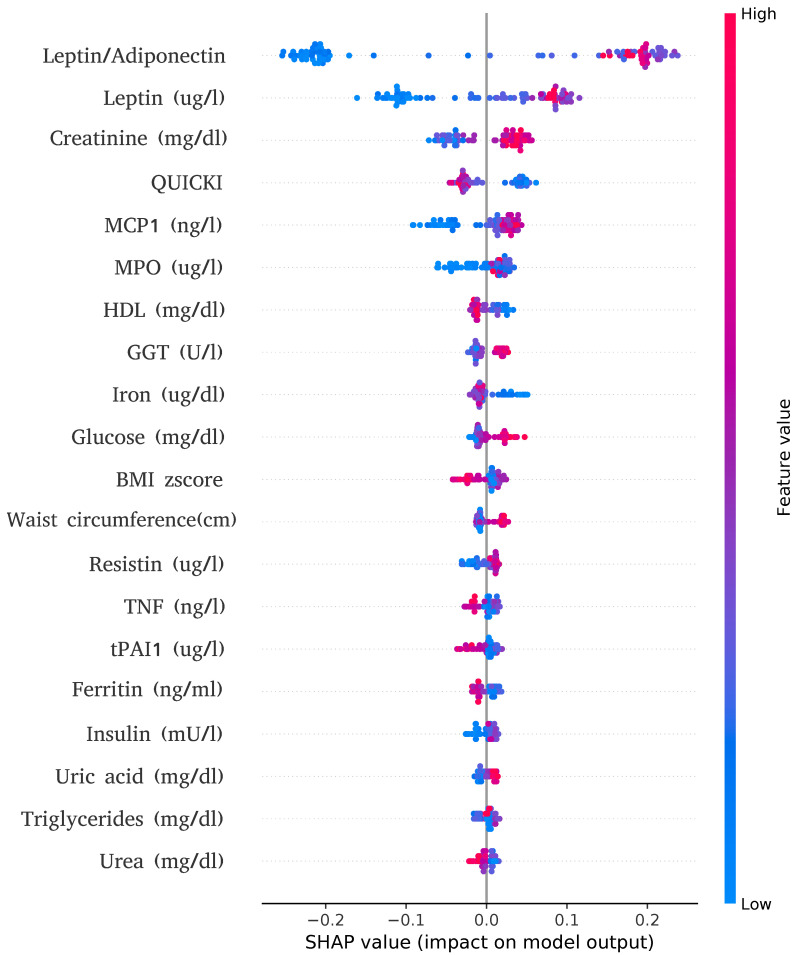
SHAP dot plot. The 20 most important attributes of the biochemistry datasets are displayed according to their overall importance. Each dot represents the value of a sample for a given attribute, and its SHAP value is its contribution to the model’s output for a specific sample. Dot colors indicate if the value of the sample for the attribute is high (red) or low (blue).

Given these results, our next step is to uncover the mechanisms behind the XGBoost model trained with the biochemistry dataset by undersampling the majority class. We used SHAP to uncover the mechanisms behind classifier predictions using the XGBoost algorithm, which performed slightly better in the distinct omics layer showing robust behavior. From the three possible models, we chose the one that showed the best average result, taking into account the accuracies in both classes (as can be reflected in the values of the G-mean measure). Figure 3 illustrates the top 20 variables for the biochemistry dataset ranked according to their relevance in the model’s output. Each dot represents the impact on the model’s output for a specific dataset attribute and sample. Our model generates an output between 0 (no-IR) and 1 (IR), and intermediate values are rounded to 0 or 1, so that positive SHAP values can be interpreted as pushing the model toward predicting IR while negative values push in the opposite direction. Taking this into account, we can determine what weight (positive or negative) attribute the dataset has in the final output of the algorithm. The dotted colors also provide useful information: red dots indicate high values for that attribute and blue colors mean the values are low. Interestingly, the vertical axis of the SHAP zero value usually separates the red and blue colors, which suggests that, depending on the attribute, high values push the algorithm toward making a decision while blue dots do the opposite.

**Figure 4:**
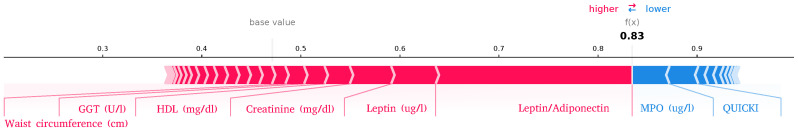
SHAP plot for a specific sample. This plot shows how the classifier output (0.83) is calculated from the different attributions (positive or negative) of the different predictors. Only the most important attributes are labeled for clarity.

Next, we will move from a general vision of the dataset to a more specific one. Figure 4 shows the influence of the most important attributes for a particular individual. The classifier score was 0.83, which means that it is predicted as IR because some validated attributes, such as the leptin/adiponectin ratio, creatinine (mg/dL), and HDL (mg/dL) are key in pushing the algorithm toward that decision, while MPO (μg/L) and QUICKI reduced the risk of IR. The base value represents the mean of the predictions for the whole dataset and is the starting point from which the attributions of the different predictors are added or subtracted.

## 4. Discussion

### 4.1. Main Challenges That Are Usually Faced in Omics ML Predictive Modeling

Human research faces a range of difficulties (e.g., patient recruitment; access to invasive biopsies and high costs), which directly result in studies with relatively low sample sizes. This issue is evident in the context of omics data, where there are millions of variables measured that massively increase the rate of false-positive discoveries (i.e., the curse of dimensionality). In the context of ML predictive modeling, low sample sizes and a huge search space have direct effects on the performances of the models built with omics data, leading to increases in computational burden and overfitting. For these reasons, it is essential to perform feature-selection steps prior to model training. There are several ways to perform feature selection, and choosing which features to use depends on the characteristics of the data and the research problems to be evaluated. Another common challenge in human research is the high presence of unbalanced designs in which one class is over-represented in relation to the other. This often occurs in a setting where the disease under study is not frequent and the recruitment of patients is complicated. As is often the case with low sample sizes and high-dimensionality problems, the class imbalance directly affects the ML predictive models by inducing overfitting. Multiple solutions have been proposed to face this problem (e.g., undersampling and oversampling) depending on the characteristics of the samples under consideration. In our case study, we demonstrated that the undersampling solution is one of the most recommended for most human contexts since it avoids introducing additional noise into the data. Another issue of importance when dealing with human data is the strong variability that exists between subjects. To address this, the development of accurate and valid experimental designs that minimize sources of bias (such as the randomization of subjects, the gender and age balance between recruitment centers, and the control of the batch effects) is of utmost importance. Moreover, the validation of findings in an external population to ensure the reliability of predictive models is essential. To this end, in cases where it is not possible to recruit additional patients, several iterative validation solutions based on cross-validation methodologies are available [27].

Several particularities should be highlighted when focusing on the characteristics of omics data. This is due to the need to apply different preprocessing procedures for each molecular layer, which is inherent to each platform. In all omics analyses, there is some background noise or an unwanted source of variability that is associated with technical laboratory procedures. This heterogeneity is, therefore, not related to the biological issue being studied and must be removed from the analysis. Background noise due to technical procedures usually differs not only between different types of omics but also between the different technological platforms normally employed for the analysis of the omics (i.e., intra- and inter-omics variability).

GWAS refers to any observational study of a genome-wide set of genetic variants or SNPs in different individuals to see if any variant is associated with a trait. GWASs are evaluated using microarrays and are subject to several problems: erroneous genotype call assignments due to poor quality DNA samples, poor DNA hybridization to the array, poorly performing genotype probes, and sample mix-ups or contamination. Although currently available GWAS platforms map many SNPs (500,000 SNPs), there are still many unmeasured variants of interest for disease prediction that could be imputed using appropriate procedures [15]. In order to deal with these and other problems, quality control filters are usually applied in GWAS research (e.g., assessing the absence of SNPs and individuals, evaluating sex discrepancies according to sex chromosomes, filtering using minor allele frequencies, controlling Hardy–Weinberg equilibrium, heterozygosity, and population stratification). Another particularity involving genomics data is the existence of a linkage between SNPs, which means that some groups of SNPs are inherited in blocks (i.e., their minor alleles are inherited as a complete allelic phase). These SNPs are redundant for predictive purposes and a previous pruning step must be performed before passing GWAS data to a ML model [14].

In EWASs, the DNA methylation (DNAm) status across the whole genome is interrogated at the CpG level. For each molecule of DNA in a single cell, DNAm is a binary entity, in that at any cytosine it is either present or absent. However, as DNAm studies profile either bulk tissues—comprising multiple cell types—or a population of purified cells, DNAm measurements for CpGs are always reported as continuous values representing the proportion of methylated CpGs for the DNA position. Methylation levels are commonly reported as either β values or M-values (see Equations (1) and (2); as well as Figure 2). M-values have more robust statistical properties, and for that reason, they are preferred in ML tasks to β values, which have better biological interpretations and are often used to visualize data. A detailed comparison of M-values and β values is available elsewhere [22].

As with GWAS, EWAS data are also subject to many sources of unwanted variability, some of them are derived from the microarray nature of the analytical platforms, detection errors, the existence of cross-reactive probes, the need for special treatment of probes located on sex chromosomes, or the need to normalize raw fluorescence intensity signal data to address within-subject and between-subject variabilities. Regarding normalization processes, although there is no single method that is universally considered best, the functional normalization method is the most appropriate for datasets with overall differences in methylation between different tissue types [39], and the BMIQ method is considered a golden standard for dealing with datasets where large differences in terms of DNAm between samples are not expected (e.g., when all samples derive from the same tissue) [21].

The fact that EWASs analyze a mixture of cells in a tissue is an important issue as, in some cases, tissues are infiltrated by other cell types or are so heterogeneous that they might confound the findings. In blood sample types, which are most commonly analyzed in EWAS, there is an important part of variability that comes from the proportions of white cells present in each individual. Therefore, and especially when dealing with diseases with inflammatory components, as with obesity, it is extremely important to correct the findings for the proportion of white cell types presented by each subject, as it might affect the DNAm findings and, thereby, confound the effects of DNAm on the disease. This is usually resolved in EWAS using the Houseman procedure, which deduces the proportion of white cell types in each subject. Then, the estimated proportions can be included as confounding variables in the models [40].

Beyond the aforementioned technical sources of variabilities associated with each technology, there are also other particularities that affect data pre-processing and that are of particular importance when one wants to predict an outcome. With regard to GWAS data, it is a fact that certain diseases present strong polygenetic architectures; many genes are involved in the development and progression of the disease. Associated genes often have small individual effects on the phenotype, so the accumulation of many small-effect variants constitutes a susceptibility profile. In EWASs, environmental confounders can strongly affect epigenetic patterns. For this reason, it is well-known that the findings from one study population cannot be easily extrapolated to another population [9].

### 4.2. Analysis of ML Results and Insights from the Case Study

In our study, the above considerations were extrapolated to a practical scenario aimed at analyzing the predictive ability of ML models in the development of insulin resistance in children. From the ML prediction models generated with the OneR, CART, and XGBoost algorithms, the following two conclusions can be drawn:

- Models trained using the imbalanced datasets show better sensitivity at the expense of very poor specificity, while datasets balanced during the training stage provide more consistent values for both metrics and greater generalizability to unseen data of any kind.
- When the training dataset is balanced, the biochemistry dataset provides the best results in terms of F1, G-mean, accuracy, sensitivity, and specificity, followed by EWAS and GWAS; this leads us to conclude that combining biochemistry and EWAS datasets may be a promising strategy to improve these results. As Table 2 shows, the classifiers generated by OneR and CART obtain slightly higher values for the metrics analyzed on the biochemical datasets. However, XGBoost obtains similar results for the omics and higher values for the other two omics, presenting robust behavior in all of them [36].

When studying GWAS data, and looking at the accuracy, one might think that these models classify well, but this is only true for the majority class. This is relevant because the objective of our case study was the exact opposite—to predict the minority class correctly. Despite using undersampling methods to avoid overfitting in this case, it can be observed that the classifiers constructed on GWAS data were no better than randomly assigning individuals to one class or another (area under the Roc curve or AUC ≈ 0.65). In relation to the results obtained, GWAS data did not contain useful information patterns for predictive tasks. Among other reasons, this could be attributed to the complex genetic architecture of obesity traits and the additive effects of SNPs on disease risk since there are thousands of SNPs, with small risk effects on the phenotype, which constitute a high susceptibility profile. Finally, looking at Table 2, we can conclude that the use of undersampling successfully reduced overfitting in the EWAS and biochemistry datasets.

The biochemical dataset, therefore, provided the patterns with the most useful predictive information, achieving the best values in most of the metrics. The results obtained for the biochemical dataset are not surprising since it included phenotypic, anthropometric, and clinical variables with direct implications in the development of obesity (e.g., BMI z-score, or waist circumference), which are currently used in daily clinical practices to estimate the risk of metabolic syndrome in children with obesity. Moreover, through a detailed study of attributes of the biochemistry-balanced ML model, we were able to demonstrate that those with the highest IR predictive abilities (e.g., leptin/adiponectin ratio) were strongly related to the presence of obesity. In fact, the regulation of leptin levels is one of the key factors of the disease, given its implications in the development of obesity-associated comorbidities, such as non-alcoholic fatty liver disease (NAFLD) [41,42].

In future work, other ways of encoding omics data, such as genetic, methylation, or metabolic risk scores (polygenetic risk score) should be explored. In particular, the way we select the input SNPs, and how we pass that information to the ML model are very important in genetics. As we previously mentioned, obesity and other complex traits involve a complex polygenetic architecture and it has been demonstrated that directly using individual SNPs is not the best tool for predictive purposes. Otherwise, risk scores (which could also be extended to EWAS and environmental data) are powerful tools to account for the complex structures of omics data and how to best predict long-term outcomes. With risk scores, we gather information for thousands of SNPs (or variables), thus reducing the problem of dimensionality while modeling the complex structures of omics. Similarly, performing appropriate feature selections on omics data that have small sample sizes is an unsolved task in the omics ML field. For this reason, some multivariate methods could be tested by checking their promising abilities in order to deal with omics data to reduce their high dimensionality. Another crucial issue to consider according to our results is the integration of multi-omics data with biochemical and clinical data in a single model. Despite this, such combination procedures are not always as straightforward as combining all data into the same model [27].

## 5. Conclusions

In this paper, we illustrated how to face the main challenges encountered when constructing ML predictive models with multi-omics human data. The main topics covered in this paper were as follows: a description of the main particularities of the omics data layers, the most appropriate pre-processing approaches for each source, and a collection of the best practices and tips for applying ML to these kinds of data for predictive purposes. By exemplifying the generation of predictive models using real data, we showed some of the key issues that need to be addressed in this kind of research (e.g., technical noise, biological heterogeneity, class imbalance, high dimensionality, and the presence of missing values). This paper presents a collection of the best practices and guidelines that could be extrapolated to other human diseases with complex bases (e.g., obesity). We lay the groundwork for future work by incorporating some proposals to improve the models, advocating that they are necessary according to the insights encountered.

## Figures and Tables

**Table 1:** Summary table of the three different datasets considered in this study, showing the main features considered in each pre-processing step.

	GWAS	EWAS	Biochemistry
Initial variables	651,563	866,091	48
Variables with low quality or missing values	138,626	31,184	14
	(21.27%)	(3.60%)	(15.2%)
% missing values after quality filtering	0%	0%	0.9%
Final number of variables	5,894,726	834,371	34
Final number of variables after feature selection	151	267	34

**Table 2:** Classification metrics obtained using OneR, CART, and XGBoost classifiers with/without undersampling in the different training sets.

OneR	Datasets			Datasets (Undersampling)		
**Metrics**	**GWAS**	**EWAS**	**Biochem.**	**GWAS**	**EWAS**	**Biochem.**
G-mean	0.27	0.44	0.46	0.40	0.44	**0.67**
AUC	0.48	0.51	0.54	0.42	0.44	**0.67**
F1	**0.78**	0.73	**0.78**	0.48	0.43	0.66
Accuracy	0.65	0.62	**0.67**	0.42	0.44	**0.67**
Sensitivity	**0.88**	0.76	0.83	0.55	0.45	0.62
Specificity	0.08	0.26	0.25	0.29	0.42	**0.73**
**CART**	**datasets**			**datasets (undersampling)**		
**Metrics**	**GWAS**	**EWAS**	**Biochem.**	**GWAS**	**EWAS**	**Biochem.**
G-mean	0.00	0.44	0.09	0.41	0.52	**0.66**
AUC	0.50	0.52	0.49	0.47	0.53	**0.67**
F1	**0.83**	0.75	0.82	0.55	0.52	0.62
Accuracy	**0.71**	0.63	0.69	0.47	0.51	0.67
Sensitivity	**1.00**	0.79	0.97	0.70	0.55	0.58
Specificity	0.00	0.24	0.01	0.23	0.48	**0.76**
**XGBoost**	**datasets**			**datasets (undersampling)**		
**Metrics**	**GWAS**	**EWAS**	**Biochem.**	**GWAS**	**EWAS**	**Biochem.**
G-mean	0.53	0.48	0.44	0.60	0.62	**0.64**
AUC	0.65	0.67	0.59	0.65	**0.70**	0.66
F1	0.79	**0.82**	0.74	0.59	0.59	0.64
Accuracy	0.69	**0.72**	0.62	0.60	0.62	0.64
Sensitivity	0.82	**0.91**	0.77	0.61	0.59	0.62
Specificity	0.35	0.25	0.25	0.59	0.64	**0.66**

## Data Availability

The authors affirm that all data necessary for confirming the conclusions of the article are present within the article, figures, and tables.
